# Advocating for health and climate

**DOI:** 10.2471/BLT.22.289283

**Published:** 2023-01-05

**Authors:** Jen Iris Allan

**Affiliations:** a School of Law and Politics, Cardiff University, Law Building, Museum Avenue, Cardiff CF10 3AX, Wales.

In recent years, health advocacy on climate change has gathered momentum. Until the adoption of the Paris Agreement in 2015, relatively few health-focused organizations were engaged in the United Nations Framework Convention on Climate Change (UNFCCC) annual meetings.^1^ Recently, health organizations have joined global campaigns, initiated their own programmes of work and increased their participation in the UNFCCC. However, this increased activity comes later than that of many other social advocacy groups. The late start of health advocacy on climate change might hinder advocates’ efforts to achieve health-climate actions under the Paris Agreement. 

Recent global climate change conferences buzz with activity. Tens of thousands of people gather, advocating national positions or advocacy claims. Part of the growth in participation is due to the expansion and diversification of civil society organizations’ presence. The issues these organizations raise are increasingly varied. Since the mid-2000s, organizations and movements representing social issues expanded their presence in the conferences and brought new tactics, such as protests.^1^^,^^2^ These advocates linked gender, indigenous rights, justice and labour issues, among others, to climate change. In turn, the Paris Agreement’s preamble recognizes the “right to health, the rights of indigenous peoples, local communities, migrants, children, persons with disabilities, and people in vulnerable situations and the right to development, as well as gender equality, empowerment of women and intergenerational equity.”^3^ The UNFCCC has ongoing programmes of work related to gender, indigenous people and a just transition for workers.

However, health does not have a similar institutional foothold in global climate governance. Here, I outline some of the challenges health advocates have faced mobilizing in the UNFCCC and trace the growing cohesion in the health-climate advocacy community and its messaging. Yet, the ability of health advocates to influence the global negotiations may be limited. National and transnational efforts, in coordination with environmental groups, may be more promising routes for those wishing to advance health messaging in the context of climate change.

## Health advocacy

### At the UNFCCC

**Fig. 1:**
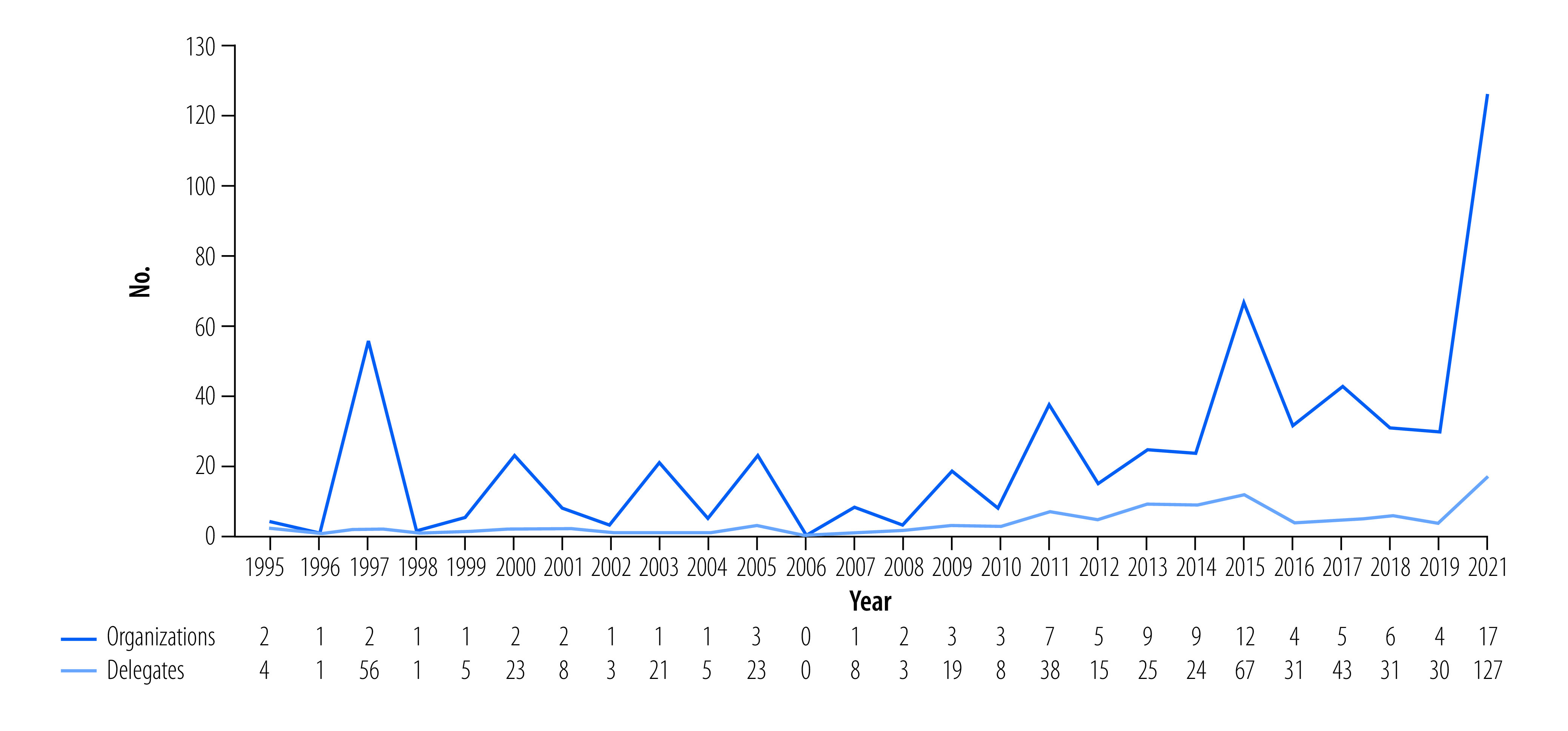
Participation of health-focused NGOs in United Nations Climate Change Conference of the Parties, 1995–2021

Health advocacy at the UNFCCC has met several challenges, particularly in terms of building a cohesive group with common messaging. But the uptick in activity (Fig. 1) among health nongovernmental organizations is significant. Activity shows that a build-up took place during the Paris Agreement negotiations (2013–2015) and then another mobilization for Glasgow (2021). Post-Paris, fewer organizations attended, but those participating sent larger delegations. A core group of organizations are devoting more resources to participate in climate Conference of the Parties.^4^

NGO: nongovernmental organization.

Notes: Data updated from Allan (2020),^1^ using official United Nations Climate Change Conference participation lists. There was no COP in 2020 due to the coronavirus disease 2019 pandemic.

Health organizations traditionally struggled to find a common message linking climate change and health. Organizations advanced different frames – which highlight certain aspects of these linkages over others. Yet, speaking with one voice would have conferred greater benefits.^5^ Some of these messages highlighted adaptation, joining the World Health Organization (WHO) in its focus on how climate change will impact public health.^1^ This frame facilitated some wins. The Nairobi Work Programme, a UNFCCC body that aims to assist developing countries (according to the Paris Agreement) in understanding and assessing climate impacts, includes health as a thematic area.^6^

A second frame highlights how reducing emissions improves health. This co-benefits frame is outlined in the World Medical Association’s resolution on a climate emergency that urges national governments to work to deliver carbon neutrality by 2030 to minimize the life-threatening impacts of climate change on health.^7^ This frame does not offer new solutions to climate change, but provides yet another motivation for action. The UNFCCC negotiations are centred on several categories of action: mitigation, adaptation, loss and damage, and support to developing countries, among others. No area of negotiations addresses why action is necessary; therefore, there is no easily identifiable institutional space for the co-benefits frame to resonate.

Recent statements indicate that a wider group of health organizations is equally articulating both frames. For example, a closing statement to the plenary of the UNFCCC meeting in Bonn in 2022 put forward four recommendations.^8^ First, phase out fossil fuels and fossil fuel subsidies as a public health imperative (the co-benefits frame); second, the Global Goal on Adaptation should protect the health of populations (the adaptation frame); third, establish a permanent discussion on food systems, including both mitigation and adaptation (both frames); and fourth, include health benefits and co-benefits of climate mitigation and adaptation across sectors in the Global Stocktake (both frames).

The statement was delivered on behalf of nine organizations and the wider public health stakeholder community. Established in 2019, the WHO-Civil Society Working Group for Action on Climate Change and Health aims to foster a strong and sustained health voice at both national and international levels. The working group has 21 members, including the two co-chairs, WHO and the Global Climate and Health Alliance, which has over 100 members.

Further institutional limits to what health advocates can expect from the UNFCCC may exist. Parties adopted the Paris Agreement and agreed on its operational rules. Periodic reviews of aggregate progress in the Global Stocktake will take place every five years, with the first stocktake concluding in 2023. The Paris Agreement is in implementation mode, and countries may be unwilling to introduce new issues or areas of work. Time-limited opportunities may present themselves, such as the dialogue on the Global Goal on Adaptation concluding in 2023. Strategically, the UNFCCC does not seem to present many opportunities to influence its rules to include health. The Paris Agreement requires countries to submit or update nationally determined contributions every five years; the next submission round is 2025. The content of these contributions is largely up to the countries. Working at the national level could help incorporate health concerns into more contributions and, more importantly, into national policy and action.

### Outside the UNFCCC

Activities at national and transnational levels may present better opportunities for influencing policy. Extinction Rebellion is a climate movement that has gained global recognition for its civil disobedience tactics. Doctors for Extinction Rebellion has introduced a new climate-health frame, which is that as trusted professionals, doctors can help build public support for climate policies. On their website, they state that because of the public’s regard for the medical profession, backing from doctors would increase political support for the cause, which could empower parliamentarians to act.^9^ This frame relies on the authority and trust held by medical professionals to present another reason to act on climate change.

Other health advocates are working outside the UNFCCC with transnational environmental movements. Support has been growing in the health sector for divesting from fossil fuels.^10^ Following *The BMJ*’s call to remove investments from fossil fuels, in a similar way as they divested from the tobacco industry, 13 medical associations and organizations have committed to divest.^4^ The health community has joined the broader fossil fuel divestment movement, which aims to raise awareness of climate change, highlight the role of fossil fuel companies and create uncertainty among investors.^11^

A second initiative is the fossil fuel non-proliferation treaty, which the Global Climate and Health Alliance supports. Behind the call is a network of 400 civil society organizations from around the world.^1^ Drawing parallels with the nuclear non-proliferation treaty’s pillars of non-proliferation, disarmament and peaceful use, the movement proposes a series of legal actions that will leave much of the world’s fossil fuel reserves in the ground.^12^

## The way forward 

For those interested in climate advocacy, strategic engagement is important. Although growing, health advocates are still a small cohort challenged by limited time and few resources. Some in the health community might be more interested in engaging in areas more directly in their control, from water sanitation to disease prevention. Cohesion will help share information and resources among those interested, but institutional barriers to action remain.

At the global level, there may be little reason to engage. Opportunities in the UNFCCC are declining as countries focus on implementing the Paris Agreement, which includes only a preambular reference to health, among other human rights.

Action can be effective at the national level. Responsibility for systemic change lies with national governments and large corporations. Many health associations and organizations are nationally based and well regarded. They could be well positioned to explain how climate action could also protect public health, whether through reducing emissions and air pollution or planning to reduce illness and injuries exacerbated by climate change. The health message holds great potential, particularly when articulated by trusted professionals. For those who are interested, difficult decisions regarding the most effective forum and framing for that message will have to be made.
